# The Principles and Practice of Modern Surgery

**Published:** 1860-10

**Authors:** 


### BOOK AND PAMPHLET NOTICES.

The Principles and Practice of Modern Surgery. By Robert Druitt. Published by Blanchard & Lea, Philadelphia.

This is an admirable edition, just prepared from the last English edition. It contains one-third more matter than the previous edition, and has received a number of valuable additions from the American edition. The chapters on the Ophthalmoscope, on Ovariotomy, on Vesico-Vaginal Fistula, on the radical Cure of Hernia, and on the Excision of the Knee Joint, are especial improvements upon former editions. Not the least of the practical advances made by the author, is the recognition of the fact that Pymæia is but a result of Erysipelas, and is a preventable accident, so that very much of the mortality after hospital operations is unnecessary.

These views were promulgated by the writer of this notice several years ago, and it is gratifying to see that other Surgeons are arriving at the same results. Meanwhile, the practical result of this advance, is, that in Mercy Hospital, Chicago, no patient has died or lost a limb from traumatic erysipelas, and no case of pyæmia occurred since the writer had charge of the Surgical wards.

